# Constitutive Vagus Nerve Activation Modulates Immune Suppression in Sepsis Survivors

**DOI:** 10.3389/fimmu.2018.02032

**Published:** 2018-09-06

**Authors:** Minakshi Rana, Yurong Fei-Bloom, Myoungsun Son, Andrea La Bella, Mahendar Ochani, Yaakov A. Levine, Pui Yan Chiu, Ping Wang, Sangeeta S. Chavan, Bruce T. Volpe, Barbara Sherry, Betty Diamond

**Affiliations:** ^1^Center for Autoimmune, Musculoskeletal and Hematopoietic Diseases, The Feinstein Institute for Medical Research, Manhasset, NY, United States; ^2^Center for Immunology and Inflammation, The Feinstein Institute for Medical Research, Manhasset, NY, United States; ^3^SetPoint Medical Corporation, Valencia, CA, United States; ^4^Center for Biomedical Science, The Feinstein Institute for Medical Research, Manhasset, NY, United States

**Keywords:** innate immune response, sepsis survivors, vagus tonic activity, CD4^+^ ChAT^+^ T cell, TNFα

## Abstract

Patients surviving a septic episode exhibit persistent immune impairment and increased mortality due to enhanced vulnerability to infections. In the present study, using the cecal ligation and puncture (CLP) model of polymicrobial sepsis, we addressed the hypothesis that altered vagus nerve activity contributes to immune impairment in sepsis survivors. CLP-surviving mice exhibited less TNFα in serum following administration of LPS, a surrogate for an infectious challenge, than control-operated (control) mice. To evaluate the role of the vagus nerve in the diminished response to LPS, mice were subjected to bilateral subdiaphragmatic vagotomy at 2 weeks post-CLP. CLP-surviving vagotomized mice exhibited increased serum and tissue TNFα levels in response to LPS-challenge compared to CLP-surviving, non-vagotomized mice. Moreover, vagus nerve stimulation in control mice diminished the LPS-induced TNFα responses while having no effect in CLP mice, suggesting constitutive activation of vagus nerve signaling in CLP-survivors. The percentage of splenic CD4^+^ ChAT-EGFP^+^ T cells that relay vagus signals to macrophages was increased in CLP-survivors compared to control mice, and vagotomy in CLP-survivors resulted in a reduced percentage of ChAT-EGFP^+^ cells. Moreover, CD4 knockout CLP-surviving mice exhibited an enhanced LPS-induced TNFα response compared to wild-type mice, supporting a functional role for CD4^+^ ChAT^+^ T cells in mediating inhibition of LPS-induced TNFα responses in CLP-survivors. Blockade of the cholinergic anti-inflammatory pathway with methyllcaconitine, an α7 nicotinic acetylcholine receptor antagonist, restored LPS-induced TNFα responses in CLP-survivors. Our study demonstrates that the vagus nerve is constitutively active in CLP-survivors and contributes to the immune impairment.

## Introduction

Sepsis is a potentially lethal illness, triggered in many cases by a gram-negative bacterial infection (1, 2). It is among the top 10 causes of death in the United States today, with over 750,000 new cases being diagnosed each year and resulting in 215,000 deaths annually (3). Marked improvements have been made in decreasing acute mortality in sepsis, but five-year survival rates have remained unchanged, irrespective of the age at which a sepsis episode occurs (4–7). Numerous clinical studies have correlated poor long-term prognosis in sepsis survivors with both dysregulated immune cell numbers and function and nosocomial infections, but the underlying mechanisms that drive these phenomena are not fully understood (8–11). The systemic inflammatory response that occurs during acute sepsis is balanced by an anti-inflammatory response resulting in a prolonged and profound state of immunosuppression, referred to as a compensatory anti-inflammatory response syndrome (12). Since many deaths of sepsis survivors are secondary to infection, the altered immune response appears to be of clinical significance (13). The identification of factors and pathways that contribute to immune dysfunction in sepsis survivors remains an area of active study (14). A contributing factor is believed to be profound alterations in monocyte/macrophage effector cell function that result in failure to appropriately respond to pathogen challenge with a robust inflammatory cytokine response (13, 15). Patients surviving sepsis often exhibit immune anergy, which increases their susceptibility to infection (16–18). The underlying mechanisms that contribute to immune anergy and impaired inflammatory responses in sepsis survivors remain unknown and are the focus of this study.

The inflammatory reflex is a neural circuit in which efferent signals transmitted through the vagus nerve regulate inflammatory cytokine production (19). In acute sepsis, the inflammatory reflex regulates cytokine production, thereby preventing potentially damaging inflammation (20). Our previous work has demonstrated that the inflammatory reflex inhibits LPS-induced TNFα production in the spleen by a mechanism requiring neural activation of acetylcholine-producing T cells (CD4^+^ ChAT^+^ T cells) leading to the release of acetylcholine, which signals through the α7 subunit of the nicotinic acetylcholine receptor (α7nAChR) expressed on cytokine-producing macrophages (21). CD4^+^ ChAT^+^ T cells are required for the inhibition of cytokine production by vagus nerve stimulation. Thus, action potentials originating in the vagus nerve and transmitted through the splenic nerve regulate CD4^+^ ChAT^+^ T cells, which in turn produce the neurotransmitter, acetylcholine, that is required to control innate immune responses (21).

In the current study, we demonstrate that in a cecal ligation and puncture (CLP) mouse model of polymicrobial sepsis-mediated by gram-negative bacteria, the vagus nerve is constitutively active in CLP-survivors, impairing their ability to respond to subsequent challenge by LPS. The reduction in LPS-induced TNFα observed in sepsis surviving mice can be partially restored by vagotomy, which severs the vagus nerve. We further demonstrate that stimulation of the vagus nerve has no effect on LPS-triggered TNFα responses in CLP-surviving mice, suggesting that there is already maximal vagus nerve activity. The constitutive activation of the vagus nerve in CLP-surviving mice was associated with an increased frequency of CD4^+^ ChAT^+^ T cells.

## Materials and methods

### Mice

Six-eight week old male BALB/c mice were purchased from Charles River Laboratories (Wilmington, MA, USA), and C57BL/6J and CD4 KO (B6.129S2-Cd4tm1Mak/J) mice were purchased from Jackson Laboratories (Bar Harbor, ME, USA). ChAT-EGFP (B6.Cg-Tg (RP23-268L19-EGFP) 2Mik/J) mice were bred at the Feinstein Institute for Medical Research. All mice were housed under specific pathogen-free conditions at room temperature (22°C) and a 12 hr light-dark cycle with access to chow diet and water. Mice were grouped randomly and assigned to a specific treatment. Sample sizes are indicated in figure legends. This study was carried out in strict accordance with recommendations in the Guide for the Care and Use of Laboratory Animals of the National Institutes of Health. The protocols were approved by the Institutional Animal Care and Use Committee and the Institutional Biosafety Committee of the Feinstein Institute for Medical Research.

### Cecal ligation and puncture and endotoxin challenge

For induction of polymicrobial sepsis, animals were subjected to CLP, which induces a lethal form of peritonitis associated with 30–50% mortality (22, 23). Briefly, CLP was performed under isoflurane anesthesia, and the cecum was isolated and ligated below the ileocecal valve and then punctured once with a 22-G needle. Approximately 1 mm of feces was extruded, the cecum was placed back to the abdominal cavity, and then the abdominal muscle layer was closed with 6.0 DemeSilk sutures followed by skin layer closure with medical-surgical clips. In control-operated (control) mice, the cecum was exposed but no ligation or puncture was performed. Both CLP and control mice received one dose of antibiotics (imipenem/cilastatin, 0.5 mg/kg diluted in a 0.9% saline solution) as a part of the resuscitation fluid (total volume of 0.5 mL/mouse, s.c.) and a single dose of sterile saline (0.5 mL/mouse, s.c.). Animals were monitored daily for survival and evaluated using the Mouse Grimace Scale twice a day for the first 3 days followed by once a day for up to 7 days after surgery. Methyllycaconitine (MLA) (24) (4 mg/kg; Sigma-Aldrich, St. Louis, MO, USA) was intraperitoneally administered 30 min prior to LPS-challenge in CLP-survivors. Endotoxin (LPS 4 mg/kg from *Escherichia coli*, 0111: B4; Sigma-Aldrich, St. Louis, MO, USA) was injected intraperitoneally. Blood and spleens were harvested at 90 min after LPS administration. Blood was collected via cardiac puncture and serum was processed within 60 min of collection by centrifugation for 15 min at 3,000 rpm and 4°C. Serum samples were stored at −80°C until analysis. Spleens were stored in cold-PBS on ice until cell isolation. TNFα concentrations in serum were measured by a mouse Proinflammatory 7-plex assay following the manufacturer's protocol (Meso Scale Discovery, Rockville, MD, USA). Plates were analyzed on the MS2400 Imager (Meso Scale Discovery). Data were quantified by fitting to a standard curve using Meso Scale Discovery Workbench software using default parameters.

### Cell isolation

Spleen cells were analyzed by flow cytometry following the established protocol at our laboratory. In brief, splenocytes were isolated by crushing spleen through 70 μm strainer in ice cold-PBS without calcium or magnesium with a 1 mL syringe plunger. Cell pellets were resuspended in red blood cell lysis buffer (BioLegend, San Diego, CA, USA) for 5 min at room temperature, and washed twice with 2% fetal bovine serum (FBS) containing PBS and counted.

### *ex vivo* LPS stimulations

Single-cell suspensions of splenocytes (6 × 10^6^ cells/mL) were cultured in flat-bottomed 96-well plates for 24 h in 200 μL RPMI medium supplemented with 10% FBS, 100 U/ mL penicillin and 100 μg/mL streptomycin (Gibco, Gaithersburg, MD, USA). Cells were cultured in medium alone or in medium containing LPS from E. coli (100 ng/mL; Serotype R515 (Re) TLR grade; Enzo, Farmingdale, NY, USA). Supernatants from cultured splenocytes were stored at −80°C until analysis.

### Flow cytometry

For flow cytometry, one million cells per sample were first blocked with Fc block (Rat anti-mouse CD16/CD32, BD Biosciences, San Jose, CA, USA) for 5 min at room temperature. Cells were then incubated with phycoerythrin (PE)-Cy7-conjugated rat anti-mouse CD11b (BD Biosciences); fluorescein isothiocyanate (FITC)-conjugated rat anti-mouse Ly6C (BD Biosciences); allophycocyanin (APC)-conjugated rat anti-mouse CD62L (BioLegend); PE-conjugated rat anti-mouse CD4 (BD Biosciences); pacific blue-conjugated rat anti-mouse CD44 (BioLegend) antibodies and Fixable Viability Dye efluor506 (Thermo Fisher Scientific, Waltham, MA, USA) for 30 min at 4°C. The cells were fixed in 1% paraformaldehyde and kept in the dark at 4°C until analysis. Data were acquired using an LSRII flow cytometer (BD Biosciences) and analyzed with FlowJo software (Tree Star, Inc., Ashland, OR, USA).

### Immunofluorescence

Spleens were fresh-frozen with dry ice, embedded in O.C.T. compound (Tissue-Tek; Thermo Fisher Scientific), and kept at −80°C until processing. Spleen slices were cut at 10 μm thickness using a Leica3050s cryostat (Leica Biosystems Inc., IL, USA) and air-dried on glass slides. All incubations were performed at room temperature in a humidified chamber. Slides were fixed in 4% paraformaldehyde (Sigma Aldrich, St. Louis, MO, USA) for 10 min and permeabilized in 1% cytofix/cytoperm solution (BD Biosciences) for 30 min. PE-conjugated rat anti-mouse TNFα antibody (eBiosciences) was diluted (1:50 dilution) in 1% cyto/perm solution. After a 2 h incubation period, the slides were washed three times in PBS containing 0.05% Tween 20, dried and mounted in Dako fluorescence mounting medium (Santa Clara, CA, USA). Slides were observed through a Zeiss LSM880 Confocal microscope. Images were analyzed and quantified by using the ZenBlue software (Zeiss, Oberkochen, Germany).

### Vagotomy

For vagotomy experiments, vagotomy was performed under isoflurane anesthesia at 2 weeks post-CLP or control surgery. The subdiaphragmatic vagus nerve was exposed from the ventral aspect and both the ventral and dorsal branch of the vagus nerves were dissected (25). For non-vagotomized mice, the vagus nerve was gently exposed without further manipulation. Mice were administered 0.5 mL sterile saline to aid recovery from surgery. Animals were monitored for 7 days.

### Electrical stimulation of the vagus nerve

Male BALB/c mice were anesthetized with 100 mg/kg ketamine and 10 mg/kg xylazine i.p. Vagus nerve stimulation (VNS) was performed as described previously (26). In brief, a ventral midline cervical incision was made and the left carotid sheath was isolated between the sternomastoid and sternohyoid muscles. A custom-built bipolar cuff electrode (MicroProbes, Gaithersburg, MD, USA) with a silastic coated platinum-iridium wire lead and an internal diameter of 0.3 mm was held about the outside of the entire carotid sheath containing the vagus nerve. In mice without vagus nerve stimulation, the left carotid sheath was exposed, but not stimulated. Electrical pulses (biphasic, 0.75 mA current output, 0.25 ms pulse width, 10 Hz frequency, 60 s pulse train) were delivered by a custom-built stimulator (SetPoint Medical Inc., Valencia, CA, USA). Each mouse was allowed to recover from anesthesia for 3 hr and after that injected with LPS (4 mg/kg; i.p.). Mice were euthanized 90 min after LPS injection, and serum was obtained for TNFα determination.

### Statistical analysis

Statistical analysis was performed with GraphPad Prism 5 (GraphPad Inc., La Jolla, CA, USA) using the Mann-Whitney U test (two groups) or Tukey's *post hoc* one-way ANOVA (three or more groups) and *p*-values < 0.05 were considered significant.

## Results

### Constitutive activation of the vagus nerve in sepsis surviving mice impairs their ability to respond to LPS-challenge

**Figure 1:**
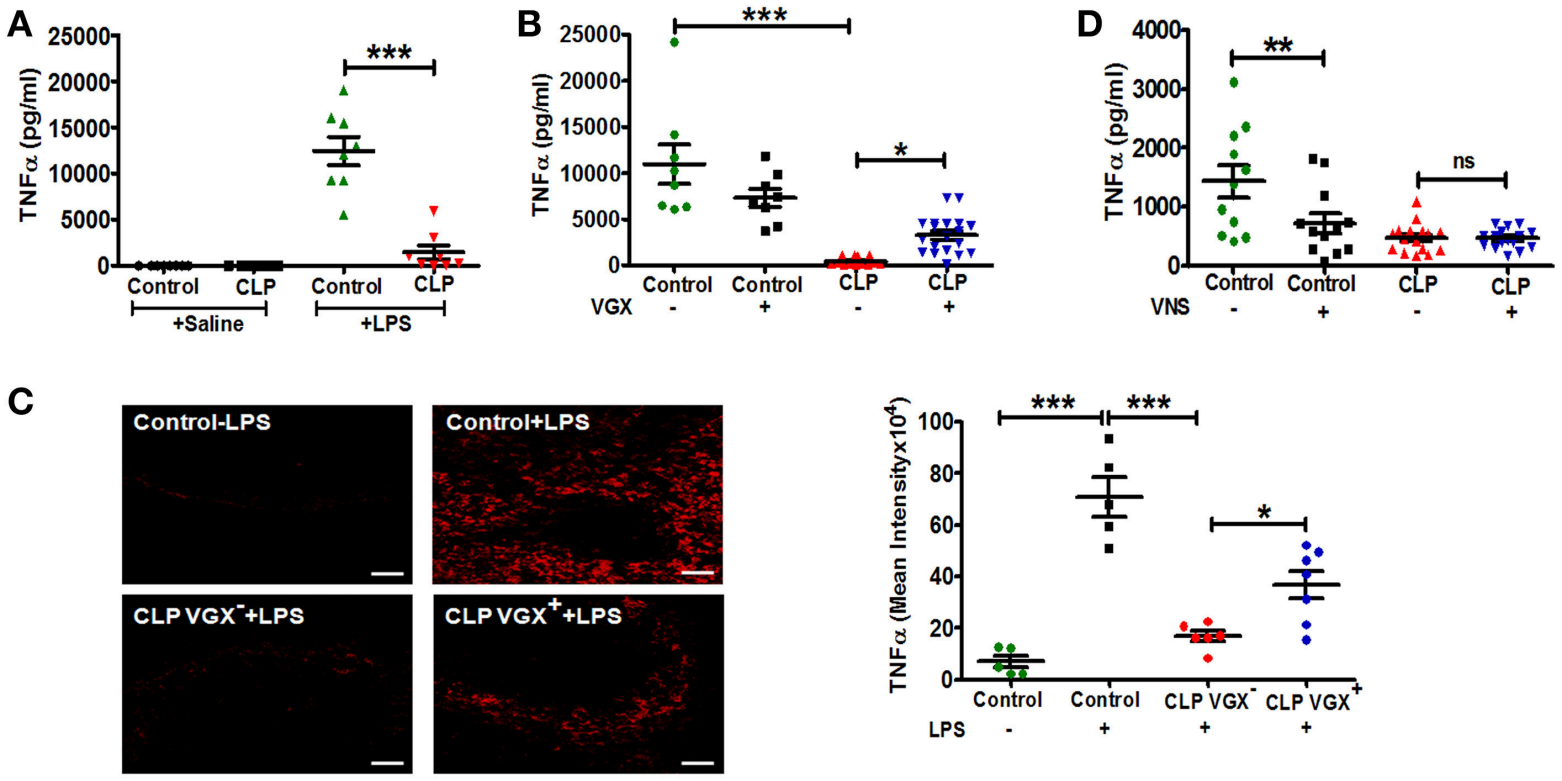
Vagotomy restores LPS-induced TNFα response in CLP-survivors. To assess the *in vivo* immune response of CLP-surviving mice to subsequent LPS challenge, BALB/c mice were subjected to control or CLP surgery, allowed to recover for 4 weeks, and then administered LPS (4 mg/kg) by intraperitoneal injection. Vagotomy (VGX) was performed 2 weeks post-CLP or control surgery. Blood and spleens were harvested 90 min after LPS administration. **(A)** Lower TNFα levels in serum of sepsis survivors in response to LPS challenge *in vivo*. (*n* = 8 mice/group). Results are the mean ± SEM from two independent experiments, Control + LPS vs. CLP + LPS ^***^p < 0.001 (Tukey's *post hoc* test). **(B)** Restoration of LPS-induced TNFα responses following bilateral subdiaphragmatic vagotomy in sepsis survivors. (*n* = 8–19 mice/group). Results are the mean ± SEM from three independent experiments, CLP VGX^−^ vs. CLP VGX^+^^*^p < 0.05 and Control VGX^−^ vs. CLP VGX^−^^***^p < 0.001 (Tukey's *post hoc* test). **(C)** Anatomical localization of splenic LPS-induced TNFα in sepsis survivors. (*n* = 5–7 mice/group). Results are the mean ± SEM from two independent experiments, CLP VGX^−^ + LPS vs. CLP VGX^+^ + LPS ^*^p < 0.05; Control without LPS vs. Control + LPS or Control + LPS vs. CLP VGX^−^ + LPS ^***^p < 0.001 (Tukey's *post hoc* test). Magnification: (20X; scale bar = 50 μm). **(D)** Mice were subjected to with or without vagus nerve stimulation (VNS; 1 min) followed by LPS injection. Serum TNFα was measured by ELISA. (*n* = 11–16 mice/group). Results are the mean ± SEM from two independent experiments, Control VNS^−^ vs. Control VNS^+^^**^p < 0.01; CLP VNS^−^ vs. CLP VNS^+^ ns = not significant (Tukey's *post hoc* test).

To confirm previous studies demonstrating a blunted innate immune response in sepsis surviving mice, we challenged control and CLP-surviving mice with LPS administered by intraperitoneal injection 4 weeks post-surgery. CLP-survivors mounted a diminished TNFα response compared to control mice (Figure 1A). To evaluate whether there was an intrinsic alteration in monocyte/macrophage responsiveness to LPS, splenocytes were isolated from control and CLP-surviving mice and stimulated with LPS *ex vivo*. Consistent with a prior report (27), splenocytes isolated from sepsis surviving mice were responsive to LPS stimulation showing increased secretion of TNFα compared to splenocytes from control mice when stimulated with LPS *ex vivo* (Supplementary Figure 1). Taken together, these findings suggested that the reduced response to LPS challenge *in vivo* reflected an inhibitory effect of cellular networks within the spleen. It is well established that the vagus nerve regulates TNFα production by macrophages in the spleen (28). Therefore, we hypothesized that the splenic microenvironment and/or altered vagus nerve activity contributes to an altered *in vivo* monocyte/ macrophage response to LPS. To evaluate the impact of the vagus nerve on the diminished TNFα production in CLP-surviving mice, CLP-surviving and control mice were subjected to bilateral subdiaphragmatic vagotomy (VGX^+^) or non-vagotomy (VGX^−^) two weeks post-CLP surgery. Two weeks after vagotomy, LPS was administered to the mice and serum TNFα levels were evaluated. We again observed significantly lower LPS-induced TNFα levels in the CLP-surviving, non-vagotomized (CLP VGX^−^) cohort compared to the control non-vagotomized (Control VGX^−^) cohort, as expected (Figure 1B). In sharp contrast, CLP-surviving vagotomized (CLP VGX^+^) mice exhibited significantly increased serum TNFα levels in response to LPS injection compared to CLP VGX^−^ mice (Figure 1B). There was no difference between control VGX^−^ mice and control VGX^+^ mice (Figure 1B). To confirm that the serum response reflects the activation of cells within the spleen, we also performed immunofluorescence analyses of the LPS-induced TNFα expression in the spleen sections from control mice, and CLP VGX^−^ and CLP VGX^+^ mice. Strong TNFα staining was observed in spleen sections from LPS-challenged control mice (Figure 1C, upper right panel) while negligible TNFα staining was observed in LPS-challenged CLP VGX^−^ mice (Figure 1C, lower left panel), consistent with differences observed in serum TNFα levels. As in the blood, TNFα immunoreactivity was partially restored in spleen sections of LPS-challenged CLP VGX^+^ compared to CLP VGX^−^ mice (Figure 1C, lower right panel). These data suggest that the vagus nerve is modulating LPS-triggered TNFα responses in CLP-surviving mice and is consistent with previous data showing that vagus nerve stimulation results in attenuated TNFα expression in LPS-challenged naive mice (28).

### Vagus nerve stimulation fails to lower LPS-induced serum TNFα levels in CLP-surviving mice

We next tested whether vagus nerve stimulation would alter LPS-induced TNFα levels in CLP-surviving mice as it is reported to do in mice that have not undergone CLP surgery. While electrical stimulation of the vagus nerve in control mice significantly (*p* < 0.01) decreased LPS-induced serum TNFα levels as expected, it had no significant effect on LPS-induced serum TNFα levels in CLP mice (Figure 1D). This finding is consistent with our hypothesis that vagus nerve signaling is constitutively active in CLP-surviving mice.

### Vagotomy does not alter the number of myeloid cells in the spleen of sepsis survivors

As previously shown (23), a marked expansion of the myeloid compartment occurs in CLP mice (Supplementary Figures 2A,B). There were significant increases in CD11b^+^Ly6C^high^ and CD11b^+^ Ly6C^low^ myeloid populations in the spleen at two (*p* < 0.001) and four (*p* < 0.01) weeks post-CLP compared to control mice (Supplementary Figures 2A,B). Similar increases in CD11b^+^Ly6C^high^ and CD11b^+^ Ly6C^low^ were observed in bone marrow and blood at 2 weeks post-CLP (data not shown). To address whether the effect of vagotomy might be due, at least in part, to changes in the number of monocyte/macrophage populations in the spleen of sepsis survivors, we quantified splenic monocytes/macrophages from CLP VGX^−^ and CLP VGX^+^ mice 4 weeks post-surgery. Vagotomy did not significantly alter the total number of spleen cells or the number of CD11b^+^Ly6C^high^ or CD11b^+^Ly6C^low^ myeloid populations in CLP mice (Supplemental Figure 2C), suggesting that the changes in serum TNFα levels observed in vagotomized mice were not the result of an expanded monocytes/macrophages population.

### Vagus nerve tonic activity increases acetylcholine-producing T cell numbers in sepsis survivors

**Figure 2:**
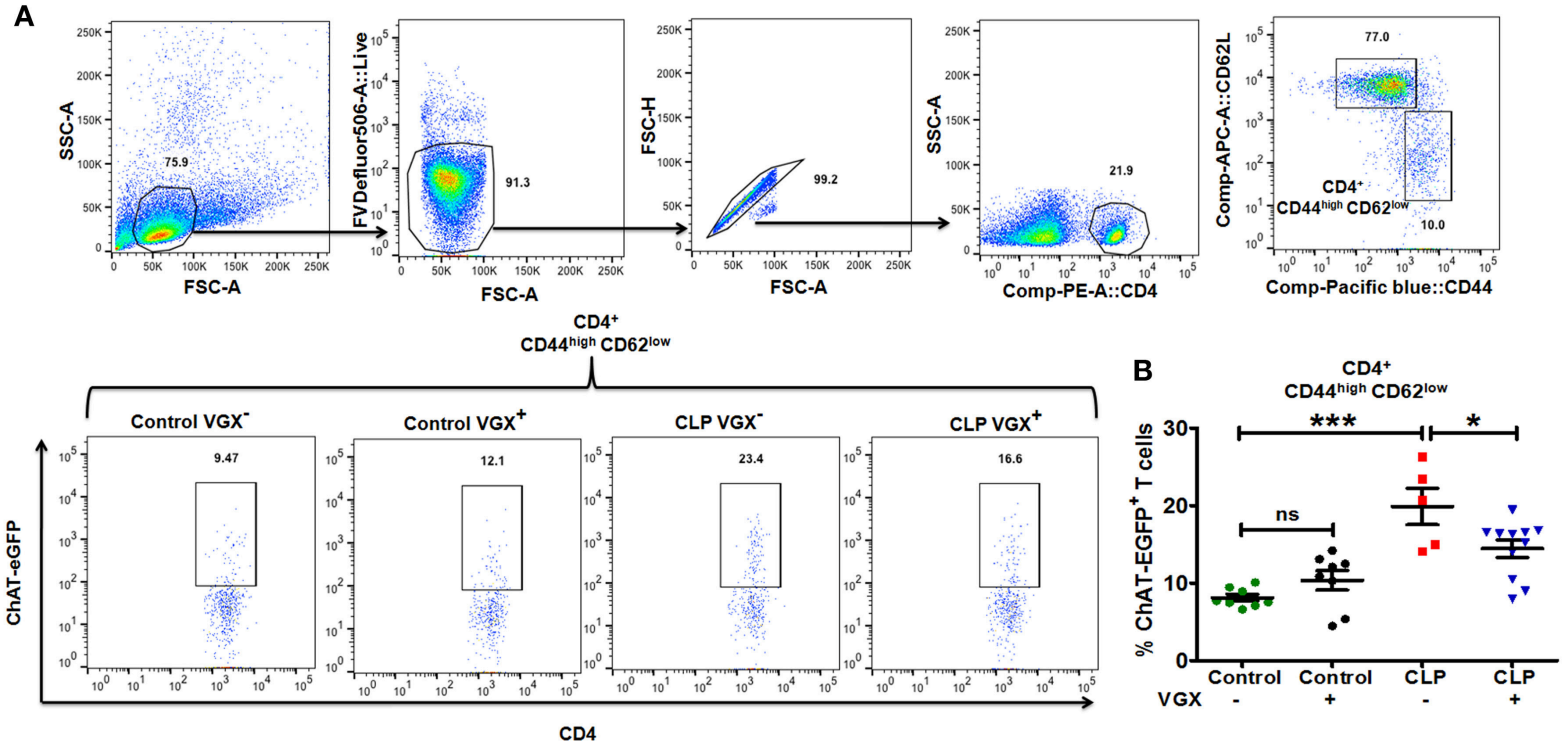
Vagus nerve signaling increases the number of ChAT^+^ memory T cells in CLP-survivors. To determine the number of ChAT^+^ T cells, ChAT-EGFP mice were subjected to control or CLP surgery. Vagotomy was performed 2 weeks post control and CLP surgery. Splenocytes isolated from CLP or control mice at 4 weeks post-surgery were stained for CD4, CD44, and CD62L and analyzed by flow cytometry. **(A)** Representative dot plots depicting the gating strategy for splenic CD44 and CD62L expression in CD4^+^ T cells and ChAT-EGFP expression in CD4^+^ CD44^high^CD62L^low^ cells. **(B)** The percentage of ChAT-EGFP^+^ cells among spleen CD4^+^ CD44^high^CD62L^low^ cells. Results represent the mean ± SEM of 5–11 mice per group from two independent experiments; CLP VGX^−^ vs. CLP VGX^+^^*^p < 0.05; Control VGX^−^ vs. CLP VGX^−^^***^p < 0.001 and Control VGX^−^ vs. Control VGX^+^ ns = not significant (Tukey's *post hoc* test).

Previous studies have shown that one mechanism by which the nervous system can regulate immune responses is through the actions of ChAT^+^ T cells that are a subset of memory CD4^+^ T cells (CD62L^low^; CD44^high^) (21). Stimulation of the vagus nerve initiates a sequence of events that culminates in the release of acetylcholine by ChAT^+^ T cells, which acts on α7 nicotinic receptors on myeloid cells to suppress LPS-induced TNFα responses (21). Based on our observation that vagus nerve stimulation failed to downregulate the *in vivo* response to LPS-challenge in CLP-surviving mice, we sought to determine whether the numbers of ChAT^+^ T cells differed in the spleens of control and CLP mice. For these cell quantification studies, we used ChAT-EGFP mice which express EGFP under the control of transcriptional regulatory elements for ChAT, the enzyme that catalyzes the biosynthesis of acetylcholine. Flow cytometry revealed that the percentage of splenic ChAT-EGFP^+^ cells among memory CD4^+^ CD44^high^CD62L^low^ T cells was increased in CLP mice compared to control mice (8.12 ± 0.43% Control VGX^−^ vs. 19.9 ± 2.36% CLP VGX^−^; SEM), and ablation of vagus signals by vagotomy in CLP mice resulted in a decrease in the percentage of ChAT-EGFP^+^ cells among memory T cells (19.9 ± 2.36% CLP VGX^−^ vs. 14.4 ± 1.11% CLP VGX^+^; SEM) (Figures 2A,B). Moreover, the numbers of splenic ChAT-EGFP^+^ cells among the memory population was also increased in CLP mice compared to control mice (Control VGX^−^ 0.145 ± 0.019 vs. CLP VGX^−^ 0.491 ± 0.143 × 10^6^ cells/ spleen; SEM) while CLP VGX^+^ mice exhibited reduced numbers of ChAT-EGFP^+^ cells (CLP VGX^−^ 0.491 ± 0.143 vs. CLP VGX^+^ 0.226 ± 0.046 × 10^6^ cells/ spleen; SEM) (Supplementary Figure 3). These observations suggest constitutive vagus nerve activation in CLP-surviving mice.

### Vagus nerve endogenous activity requires CD4 T cells to mediate inflammatory reflex

**Figure 3:**
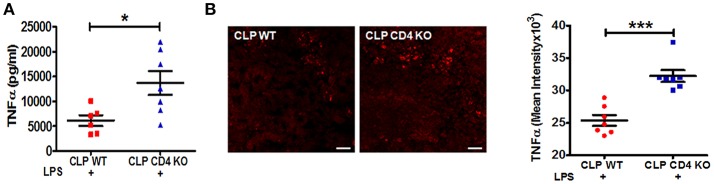
CD4 T cell-deficiency restores LPS-induced TNFα responses in CLP-survivors. To identify the role of CD4^+^ T cells in mediating inflammatory reflex, C57BL6/J WT and CD4 knockout mice were subjected to CLP surgery, allowed to recover for 4 weeks, and then challenged with LPS by intraperitoneal injection (6 mg/kg). Blood and spleens were harvested 90 min after LPS administration. Serum TNFα was measured by ELISA. **(A)** Enhanced LPS-induced serum levels of TNFα in endotoxemic CD4 T cell-deficient CLP-surviving mice. Results represent the mean ± SEM of 6–7 mice per group from one of two independent experiments; CLP WT + LPS vs. CLP CD4 KO + LPS ^*^p < 0.05. (Mann-Whitney *U*-test). **(B)** TNFα staining in spleen sections of CLP WT and CLP CD4 KO. Magnification (20X; scale bar = 50 μm). Images are representative of spleen sections from two independent experiments (*n* = 7). CLP WT + LPS vs. CLP CD4 KO + LPS ^***^p < 0.001. (Mann-Whitney *U*-test).

To confirm a functional role of CD4^+^ ChAT^+^ T cells in mediating the reduction in the LPS-triggered TNFα response, CD4 T cell-deficient and wild-type **(**WT) C57BL/6J mice were subjected to CLP surgery. LPS-triggered serum TNFα levels were significantly enhanced in CLP CD4-deficient mice compared to CLP WT mice (Figure 3A). Increased numbers of TNFα positive cells were observed in spleens of CLP CD4-deficient mice, paralleling changes observed in serum TNFα levels (Figure 3B).

### Pharmacological blockade of vagus nerve signaling restores LPS-triggered TNFα responses in CLP mice

**Figure 4:**
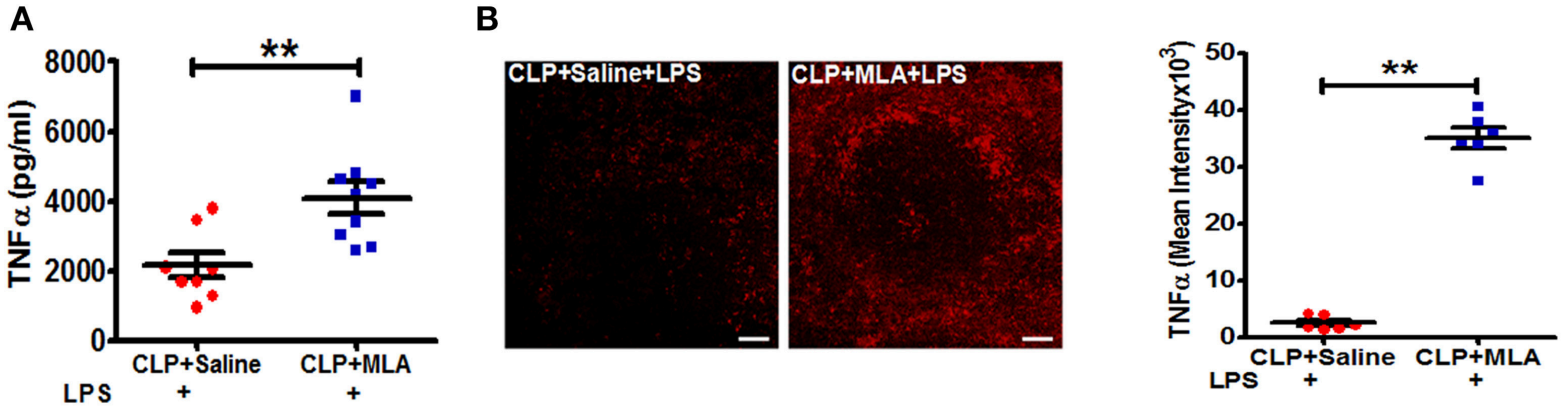
Methyllycaconitine treatment restores LPS-induced TNFα responses in CLP-survivors. BALB/c mice were subjected to control and CLP surgery, allowed to recover for 4 weeks, and then treated by intraperitoneal injection with methylcaconitine (MLA; 4 mg/kg) or vehicle (saline) 30 min prior to LPS-challenge (4 mg/kg). Blood and spleens were harvested 90 min after LPS administration. Serum TNFα was measured by ELISA. **(A)** Enhanced LPS-induced serum levels of TNFα in endotoxemic MLA-treated CLP-surviving mice. Results represent the mean ± SEM of 6–9 mice per group from two independent experiments; CLP + Saline + LPS vs. CLP + MLA + LPS ^**^p < 0.01. (Mann-Whitney *U*-test). **(B)** TNFα staining in spleen sections of CLP + Saline + LPS and CLP + MLA + LPS. Magnification (20X; scale bar = 50 μm). Images are representative of spleen sections from two independent experiments (*n* = 6). CLP + Saline + LPS vs. CLP + MLA + LPS ^**^p < 0.01. (Mann-Whitney *U*-test).

We next employed pharmacologic blockade of the α7nAChR to confirm that suppression of LPS-triggered TNFα responses in sepsis surviving mice occurs through the cholinergic pathway. CLP-surviving mice were treated with MLA, an α7nAChR antagonist (29), or vehicle at four weeks post-CLP. Pharmacologic inhibition of the α7nAChR with MLA led to a significant increase in LPS-induced TNFα levels in CLP mice compared to saline treated-CLP mice (Figure 4A). Consistent with the increase in serum TNFα levels, MLA treatment significantly enhanced the number of TNFα positive cells in the spleen (Figure 4B). Together, these data suggest that altered cholinergic tone is mediating its effects through α7nAChR.

## Discussion

Using an experimental model of sepsis, we have explored the mechanisms that trigger immune dysfunction in sepsis surviving hosts. We have demonstrated that in sepsis survivors, inhibitory signals from the brain to the immune system are chronically active. We have further demonstrated that if we surgically cut the vagus nerve that transmits these signals, or if we use drugs that block vagus nerve signaling, we can restore immune function. This study is of clinical significance as patients surviving severe sepsis exhibit persistent immune dysfunction and an inability to effectively control subsequent infections, which contributes to poor long-term outcomes. In fact, sepsis survivors have dramatically increased mortality rates within 5 years of the septic episode that are attributable, at least in part, to immune dysfunction (13, 30, 31). Several reports have identified that monocytes exposed to a septic insult or to a high dose of LPS exhibit decreased production of TNFα and other cytokines upon rechallenge with LPS (32, 33). Here we demonstrate that reduced TNFα responses following LPS-challenge in CLP-survivors are due in part to altered monocyte/macrophage function in the spleen. Paradoxically, splenic monocytes/ macrophages in CLP mice that are hyporesponsive to LPS-challenge *in vivo* generate a robust TNFα response upon exposure to LPS *ex vivo* suggesting that hyporesponsiveness *in vivo* derives from signals within the microenvironment. Previous studies have shown that vagus nerve stimulation using electrical or pharmacological approaches in models of endotoxemia and sepsis results in suppression of LPS-induced TNFα production in splenic macrophages through activation of the cholinergic anti-inflammatory pathway (28, 34, 35). The role of the vagus nerve in mediating immune suppression was confirmed in the present study as surgical ablation of vagus nerve signals by subdiaphragmatic vagotomy led to the restoration of LPS-triggered TNFα production (Figures 1B,C). Importantly, enhanced TNFα production in vagotomized CLP mice is not the result of altered numbers of inflammatory monocytes in the spleen as inflammatory monocyte numbers were equivalent in vagotomized and non-vagotomized CLP-survivors.

Studies by others have demonstrated that vagus nerve stimulation activates the adrenergic splenic nerve to release norepinephrine, which then binds to β2 adrenergic receptors expressed on CD4^+^ ChAT^+^ T cells that express choline acetyltransferase, the rate-limiting enzyme in acetylcholine biosynthesis (21, 28). In the present study, we show that the immunosuppressive effects of constitutive vagus nerve activation in sepsis surviving mice are mediated through an increased number of CD4^+^ ChAT^+^ T cells (Figure 2). Moreover, enhanced endogenous cholinergic tonic activity in CLP-surviving mice failed to attenuate TNFα production in CD4-deficient mice, suggesting that CD4^+^ ChAT^+^ T cells are indispensable to mediate the inflammatory reflex (Figure 3). Reciprocally, vagus nerve stimulation failed to suppress TNFα release in response to LPS-challenge in CLP mice (Figure 1). The failure to respond to vagus nerve stimulation might reflect an impairment in (1) nerve transduction, (2) the response of acetylcholine**-**producing T cells in the spleen to norepinephrine released by the splenic nerve or (3) the response of splenic monocytes to acetylcholine engagement of α7 receptors, or it might reflect tonic activity of the vagus nerve and an inability to exhibit a further decrease in response to vagus nerve stimulation (36). It has been shown previously that the α7nAChR is required for acetylcholine**-**mediated inhibition of TNFα release by splenic macrophages (37); the suppressive effect of vagus nerve stimulation on TNFα production is lost in α7nAChR-deficient mice (37). In this study, pharmacological blockade of α7nAChR abrogated the suppression of TNFα, confirming that the cholinergic tone in CLP mice was responsible for the attenuated response to LPS (Figure 4).

**Figure 5:**
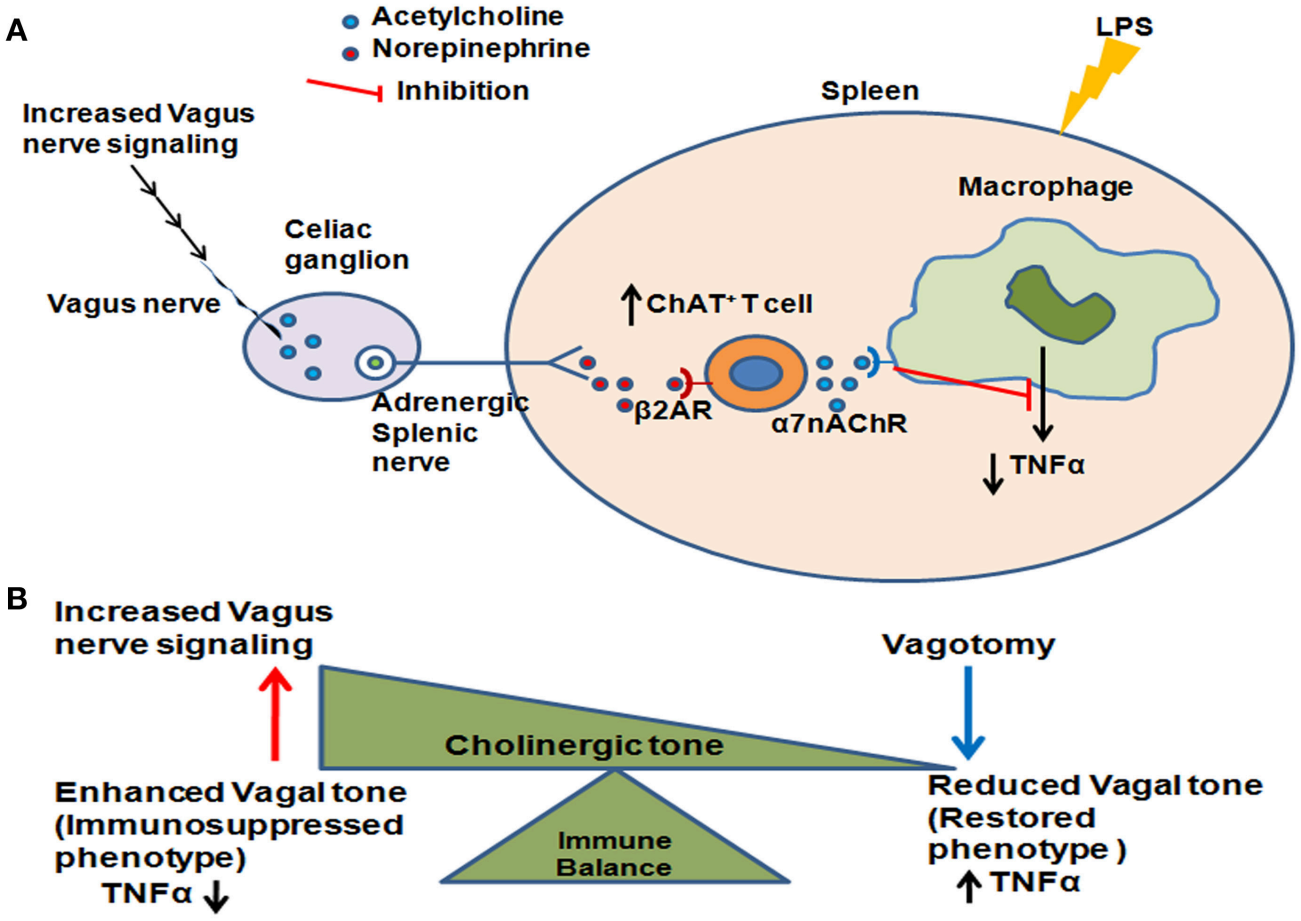
Enhanced vagus nerve signaling effects on LPS-induced TNFα in CLP-survivors. **(A)** Increased vagus nerve signaling leads to activation of the higher number of acetylcholine - producing T cells (ChAT^+^ T cells) in the spleen. Subsequent activation of α7 nicotinic acetylcholine receptor (α7AchR) on monocytes/macrophages leads to an inability to further suppress LPS-induced TNFα. **(B)** The cholinergic tone determines the immune balance, shifting between enhanced vagus nerve activation leading to an immunosuppressed phenotype and decreased vagus nerve activation leading to restored immunocompetence.

In conclusion, we have shown here that enhanced vagus nerve tonic activity leads to an immunosuppressed phenotype in sepsis survivors due to increased numbers of ChAT^+^ T cells (Figure 5). Further work needs to be done to determine whether other immune and metabolic changes observed in sepsis survivors result from constitutive activation of the vagus nerve, or whether this mechanism selectively regulates the inflammatory response. It will also be critical to confirm that these pathways are active in patients who survive sepsis. Our findings reveal that vagus nerve activation which can acutely suppress inflammation can also mediate chronic immunosuppression as is observed in sepsis surviving mice. Since vagus nerve signaling/cholinergic tone can be pharmacologically modulated, such treatments in patients who survive sepsis could represent a novel approach to prevent subsequent infections in this group.

## Author contributions

MR, YF-B, MS, BS, and BD designed the research, analyzed data and wrote the manuscript. MR, YF-B, MS, AL, MO, YL, SC, BS, and PC performed the experiments. BV analyzed the data. MR, YF-B, MS, SC, PW, BS, and BD wrote the manuscript.

### Conflict of interest statement

YL is employed by company SetPoint Medical Corporation. The remaining authors declare that the research was conducted in the absence of any commercial or financial relationships that could be construed as a potential conflict of interest.
