# Supplementary material for: Constitutive Vagus Nerve Activation Modulates Immune Suppression in Sepsis Survivors
Source: Front Immunol. 2018 Sep 6;9:2032. doi: 10.3389/fimmu.2018.02032 (PMC6135874; doi:10.3389/fimmu.2018.02032)
Supplement: Supplementary file 1 [file Data_Sheet_1.pdf]

Supplemental Material

Constitutive vagus nerve activation modulates immune suppression in sepsis survivors

Minakshi Rana[1#], Yurong Fei-Bloom[1#], Myoungsun Son[1#], Andrea La Bella[1], Mahendar Ochani[2], Yaakov A. Levine[3,4], Pui Yan Chiu[2], Ping Wang[2], Sangeeta S. Chavan[4], Bruce T. Volpe[4], Barbara Sherry[2*], Betty Diamond[1*]

[1]Center for Autoimmune, Musculoskeletal and Hematopoietic Diseases, The Feinstein Institute for Medical Research, Manhasset, NY, United States; [2]Center for Immunology and Inflammation, The Feinstein Institute for Medical Research, Manhasset, NY, United States; [3]SetPoint Medical Corporation, Valencia, California, United States; and [4]Center for Biomedical Science, The Feinstein Institute for Medical Research, Manhasset, NY, United States.

[*]Correspondence

Betty Diamond, The Feinstein Institute for Medical Research, Center for Autoimmune, Musculoskeletal and Hematopoietic Diseases, 350 Community Dr, Manhasset, New York 11030; e-mail: bdiamond@northwell.edu; Phone: 516-562-3830, Fax: 516-562-2953

Barbara Sherry, The Feinstein Institute for Medical Research, Center for Immunology and Inflammation, 350 Community Dr, Manhasset, New York 11030; e-mail: bsherry@northwell.edu; Phone: 516-562-3402, Fax: 516-562-1022

[#]MR, [#]YF-B, and [#]MS contributed equally to this study.

Supplementary Figures and Figure Legends:

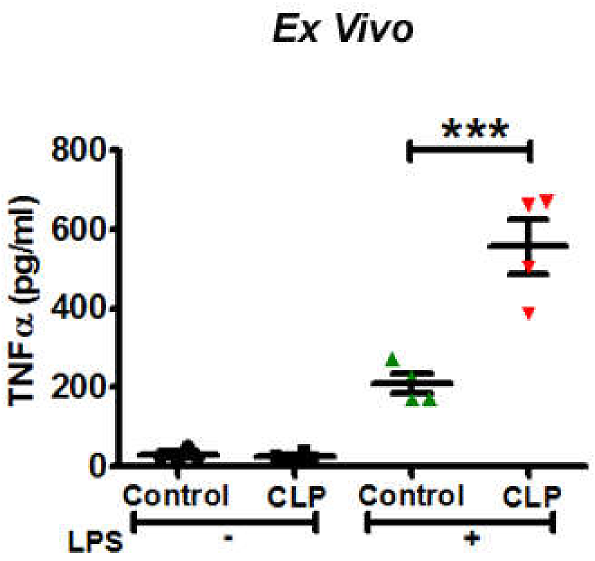

Supplementary Figure 1. Splenocytes from CLP-survivors show enhanced TNFα expression *ex vivo*. Splenocytes were isolated from control and CLP-surviving mice at 4 weeks after surgery and cultured for 24 hr with or without LPS (100 ng/ mL). Each data point represents the average of duplicate wells from a single mouse. TNFα in the culture supernatants was measured by ELISA (R&D). Values represent mean ± SEM (n=4 mice/group) from one of two independent experiments; Control+LPS vs. CLP+LPS ***$p < 0.001$ (Tukey's post hoc test).

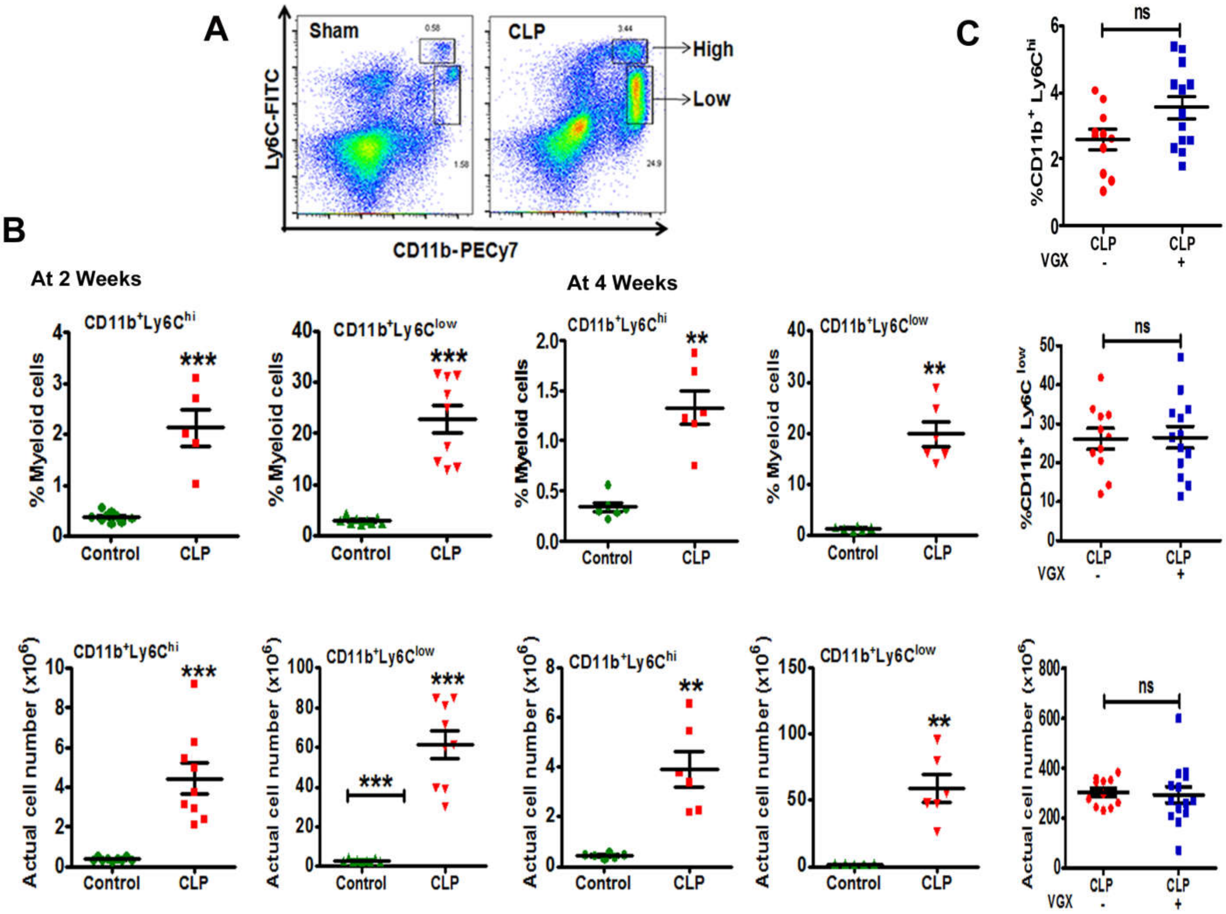

Supplementary Figure 2. CLP mice exhibit sustained expansion of the CD11b$^+$ Ly6C myeloid population in the spleen that was not altered by vagotomy. (A) Representative gating for CD11b$^+$ Ly6C^{high} and CD11b$^+$ Ly6C^{low} myeloid cells in control and CLP mice (4 weeks). (B) Percentage and numbers of CD11b$^+$ Ly6C^{high} and CD11b$^+$ Ly6C^{low} myeloid cells per spleen in control or CLP-surviving mice at 2 and 4 weeks post-surgery. (C) Vagotomy (VGX) did not alter the percentages of CD11b$^+$ Ly6C^{high} and CD11b$^+$ Ly6C^{low} myeloid cells and the actual number of total spleen cells in CLP-survivors. Values represent mean ± SEM (n=5-14 mice/group). Control vs. CLP $**p < 0.01$; $***p < 0.001$ and CLP VGX$^-$ vs. CLP VGX$^+$ ns=not significant (Mann-Whitney U test).

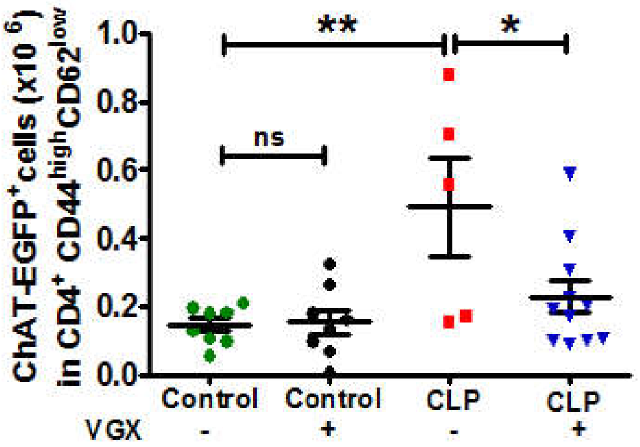

Supplementary Figure 3. Memory ChAT^{+} T cells numbers in ChAT-EGFP mice. The memory ChAT^{+} T cells in control and CLP-surviving ChAT-EGFP mice with and without vagotomy after 4 weeks surgery. Values represent mean ± SEM (n=5-11 mice/group). Control VGX^{-} vs. CLP VGX^{-} **$p < 0.01$; or CLP VGX^{-} vs. CLP VGX^{+} *$p < 0.05$; Control VGX^{-} vs. Control VGX^{+} ns=not significant (Tukey's post hoc one-way ANOVA).
